# The impact of social assistance programs on population health: a systematic review of research in high-income countries

**DOI:** 10.1186/s12889-018-6337-1

**Published:** 2019-01-03

**Authors:** Faraz V Shahidi, Chantel Ramraj, Odmaa Sod-Erdene, Vincent Hildebrand, Arjumand Siddiqi

**Affiliations:** 1 Dalla Lana School of Public Health, University of Toronto, 155 College St, Toronto, ON M5T 3M7 Canada; 2 Department of Economics, Glendon College, York University, 2275 Bayview Abe, North York, ON M4N 3M6 Canada; 3 Gillings School of Public Health, University of North Carolina, 135 Dauer Dr, Chapel Hill, North Carolina 27599 USA

**Keywords:** Social assistance, Health, Health inequalities, Income, Poverty, Social policy, Social welfare, Socioeconomic status

## Abstract

**Background:**

Socioeconomic disadvantage is a fundamental cause of morbidity and mortality. One of the most important ways that governments buffer the adverse consequences of socioeconomic disadvantage is through the provision of social assistance. We conducted a systematic review of research examining the health impact of social assistance programs in high-income countries.

**Methods:**

We systematically searched Embase, Medline, ProQuest, Scopus, and Web of Science from inception to December 2017 for peer-reviewed studies published in English-language journals. We identified empirical patterns through a qualitative synthesis of the evidence. We also evaluated the empirical rigour of the selected literature.

**Results:**

Seventeen studies met our inclusion criteria. Thirteen descriptive studies rated as weak (*n* = 7), moderate (*n* = 4), and strong (*n* = 2) found that social assistance is associated with adverse health outcomes and that social assistance recipients exhibit worse health outcomes relative to non-recipients. Four experimental and quasi-experimental studies, all rated as strong (*n* = 4), found that efforts to limit the receipt of social assistance or reduce its generosity (also known as welfare reform) were associated with adverse health trends.

**Conclusions:**

Evidence from the existing literature suggests that social assistance programs in high-income countries are failing to maintain the health of socioeconomically disadvantaged populations. These findings may in part reflect the influence of residual confounding due to unobserved characteristics that distinguish recipients from non-recipients. They may also indicate that the scope and generosity of existing programs are insufficient to offset the negative health consequences of severe socioeconomic disadvantage.

**Electronic supplementary material:**

The online version of this article (10.1186/s12889-018-6337-1) contains supplementary material, which is available to authorized users.

## Background

Decades of epidemiological research has demonstrated that socioeconomic resources such as wealth, income, and employment – often referred to as the social determinants of health – are “fundamental causes” of health inequalities [1, 2]. They are fundamental in the sense that they influence the everyday conditions, experiences, and exposures that influence health status. Put simply, those with fewer socioeconomic resources get sicker and die sooner than those higher up in the socioeconomic hierarchy. These findings have led to a broad consensus in the field of public health: social policies that shape the extent to which socioeconomic advantage and disadvantage occur in society offer the most effective, if politically contentious, strategy for reducing health inequalities [3]. Indeed, the final report of the World Health Organization Commission on the Social Determinants of Health concluded that the emphasis in public health must shift from individual-level interventions that aim to modify people’s behaviours to societal-level interventions that ameliorate their everyday socioeconomic conditions [4].

One of the most important ways that societies intervene to buffer the adverse consequences of socioeconomic disadvantage is through the provision of social assistance [5]. Social assistance refers to government programs that provide a minimum level of income support to individuals and households living in poverty. These programs lend support either in the form of direct cash transfers or through a variety of in-kind benefits (e.g. food stamps and rent subsidies). Social assistance has been shown to strengthen the purchasing power of the poor and raise their material standards of living [6, 7]. From a public health point of view, the supplemental provision of income can also enable people to avoid harmful exposures and adopt practices beneficial to their health [8]. Thus, theory predicts that social assistance programs offer an important means of protecting the health of socioeconomically disadvantaged groups and mitigating the extent of socioeconomic health inequalities [9, 10].

While there is widespread theoretical support for the role of social assistance as a policy lever with which to improve population health and promote health equity, it is unclear what the extant evidence demonstrates empirically. At the same time, there is growing concern that existing programs provide insufficient levels of protection and that such inadequacies in the social safety net produce extraordinary costs, both human and economic [11–13]. Such concern has, in some cases, prompted calls for a major overhaul of traditional social assistance schemes. In Canada, Finland, and the Netherlands, for example, governments have conducted small-scale experiments to explore the potential benefits of alternative systems of income provision, such as unconditional basic income [14]. At this critical juncture, there is a need to take stock of the extent to which existing social assistance programs are succeeding (or not) at promoting population health and health equity.

Given the clear implications of recent political developments for the health of socioeconomically vulnerable populations and the lack of clarity on the state of the extant evidence, our aim in this paper is to conduct a systematic review of peer-reviewed research that has examined the health impact of social assistance programs. We focus on programs that provide direct financial assistance rather than aid in the form of in-kind benefits. Previous reviews have evaluated the health impact of other sources of income maintenance, including food stamps [15], low-income tax credits [16], minimum wage laws [17], and unemployment insurance systems [18]. Furthermore, to avoid overlap with similar reviews in low- and middle-income countries [19], we restrict our analysis to high-income countries with well-established welfare state systems (i.e. Australia, Austria, Belgium, Canada, Denmark, Finland, France, Germany, Greece, Iceland, Ireland, Italy, Luxembourg, Netherlands, New Zealand, Norway, Portugal, Spain, Sweden, Switzerland, United Kingdom, United States). To our knowledge, this is the first systematic review to evaluate the health impact of social assistance transfers in high-income countries.

## Methods

### Search strategy

We conducted a systematic search of the literature in accordance with Preferred Reporting Items for Systematic Reviews and Meta-Analyses (PRISMA) guidelines. The search protocol was registered with PROSPERO (CRD42016048078). The search terms are listed in Table 1. We searched the following electronic databases from inception to December 31, 2017: Embase, Medline, ProQuest, Scopus, and Web of Science. We supplemented our electronic search by handsearching the reference lists of all included literature and related review articles. We restricted our search to English-language publications in peer-reviewed scientific journals. Grey literature, working papers, and peer-reviewed commentaries lacking direct empirical tests were excluded. Two authors conducted separate searches. Disagreements were resolved as a team through discussion and consensus.

**Table 1:** Search terms for the systematic review of studies examining the health impact of social assistance

Social Assistance	Health	Methods
- Social assistance	- Health inequalities	- Regression
- Social protection	- Health inequities	- Linear
- Social policy	- Health disparities	- Logistic
- Social welfare	- Health equity	- Poisson
- Social security	- Health status	- Multilevel
- Public assistance	- Mortality	- Multi-level
- Income benefits	- Public health	- Quasi-experimental
- Income supplement	- Population health	- Experimental
- Income supplementation	- Self-rated health	- Difference-in-differences
- Income maintenance		- Synthetic control
- Conditional cash		- Propensity score
- Welfare state		- Regression discontinuity
- Welfare program		- Instrumental variable
- Welfare reform		- Near-far matching
- Aid to Families with Dependent Children		- Decomposition
- Temporary Assistance for Needy Families		- Cross-sectional
- Ontario Works		- Longitudinal

The initial search yielded 2058 unique articles. Abstracts were screened to determine their eligibility for full-text review. Eligibility was determined based on four inclusion criteria: (i) reference to a social assistance program; (ii) reference to a health outcome, major risk factor for disease (e.g. hypertension and obesity), or health behaviour (e.g. smoking and diet); (iii) reference to an appropriate study population (i.e. working-age adults between 18 and 64 years of age); and (iv) reference to an empirical method of testing the health impact of social assistance or social assistance reform. We excluded studies that examined health care outcomes (e.g. health insurance coverage and physician visits) which require a distinct theoretical orientation. We also excluded studies examining maternal and child health outcomes, as these have been reviewed elsewhere [20]. Two authors marked each abstract as “Yes” if they satisfied all four inclusion criteria, “Maybe” if they satisfied two or three of the criteria; and “No” if they satisfied fewer than two of the criteria. Abstracts marked as “Yes” or with at least one “Maybe” were subject to full-text review.

### Data extraction and analytic strategy

A standardized form was used to extract relevant data from the included studies. We extracted the following information from each study: title, authors, year of publication, country, data source, sample size, main research question, study design, health outcome, and main findings. Two authors extracted the data independently. The results of the extraction were shared and discussed with the entire research team. Disagreements were resolved as a team through discussion and consensus. The extracted data was used to summarize the key features of the selected literature and synthesize the available evidence across studies. The entire research team collaborated to identify empirical patterns based on this summary and synthesis.

### Quality assessment

We assessed the quality of studies using a modified version of the Quality Assessment Tool for Quantitative Studies developed by the National Collaborating Centre for Methods and Tools. [21] We describe our method of assessment in greater detail in Additional file 1. We rated studies according to five criteria: (i) Did the study draw on a representative sample? (ii) Did the study describe the characteristics of the exposed and unexposed groups? (iii) Did the study adopt a descriptive cross-sectional, descriptive longitudinal, quasi-experimental, or experimental study design? (iv) Did the study control for important confounders such as age, gender, marital status, and education? (v) Did the study document and account for attrition (only if longitudinal). On each criterion, studies were rated as either ‘strong’, ‘moderate’, or ‘weak’. A global quality rating was derived based on whether studies had no weak ratings (‘strong’), one weak rating (‘moderate’), or two or more weak ratings (‘weak’).

## Results

### Literature search

**Fig. 1:**
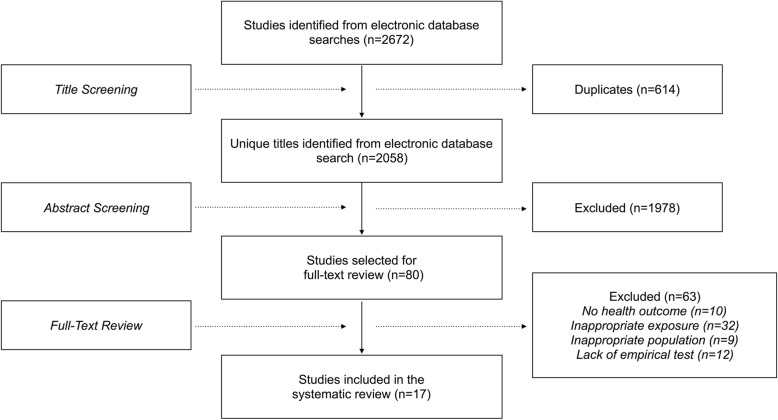
Summary of the search strategy and selection process

In Fig. 1, we summarize the results of our search strategy. Of the 2058 unique abstracts identified, eighty studies were selected for full-text review. Upon further examination, seventeen studies were found to meet our inclusion criteria. Their primary characteristics are listed in Table 2 and described in further detail below.

**Table 2:** Description of studies examining the health impact of social assistance (*N* = 17)

Authors	Country	Relevant question	Study design	Health outcome	Findings
Baigi et al. (2008) [22]	Sweden	How does the health of social assistance recipients compare to that of non-recipients?	Descriptive: Cross-Sectional	Health behavioursPsychological symptomsPhysiological symptoms	Relative to non-recipients, recipients of social assistance reported worse psychological and physiological health and worse health-related behaviours. For example, they reported higher rates of anxiety (OR 2.73, 95% CI 2.11-3.53), hand/knee pain (OR 2.33, 95% CI 1.79-3.03), and smoking (OR 4.59, 95% CI 3.56-5.93).
Basu et al. (2016) [23]	United States	How did welfare reform affect the health of social assistance recipients?	Quasi-Experimental	Health behaviours	Among low-income single mothers, welfare reform was associated with an 8.8% increase in rates of smoking (95% CI 6.8%-10.8%) and an 8.3% increase in rates of binge drinking (95% CI 4.7%-12.0%)
Butterworth (2003) [24]	Australia	How does the health of social assistance recipients compare to that of non-recipients?	Descriptive:Cross-Sectional	Psychological symptomsMental disorders	Relative to non-recipients, social assistance recipients reported higher rates of psychological symptoms (OR 2.77, 95% CI 2.38-3.24) and mental disorders (OR 1.92, 95% CI 1.64-2.26)
Butterworth et al. (2011) [25]	Australia	How does the health of social assistance recipients compare to that of non-recipients?	Descriptive:Cross-Sectional	Mental disorders	Relative to non-recipients, social assistance recipients reported higher rates of mental disorders. For example, unemployed recipients were 60% more likely (95% CI 1.02-2.54) to report a mental disorder compared to non-recipients.
Dooley and Prause (2002) [26]	United States	What is the association between a transition into social assistance and health among women?	Descriptive: Longitudinal	Health behavioursPsychological symptoms	A transition into social assistance was associated with a higher frequency of depressive symptoms (β=0.06, *p*<0.05) and higher rates of binge drinking (OR 2.06, *p*<0.05).
Ensminger and Juan (2001) [38]	United States	What is the association between baseline receipt of social assistance and later health outcomes among low-income mothers?	Descriptive: Longitudinal	Chronic conditionsPsychological symptomsSelf-rated health	Relative to non-recipients, mothers who received social assistance during young or middle adulthood reported higher rates of poor self-rated health (OR 2.51, *p*<0.05), higher rates of psychological distress (OR 1.82, *p*<0.10), and a higher frequency of depressive symptoms (β=0.17, *p*<0.10)
Ford et al. (2010) [27]	United Kingdom	Does the receipt of social assistance mitigate the adverse health consequences of unemployment?	Descriptive: Cross-Sectional	Psychological disorders	Among the unemployed, recipients of social assistance reported higher rates of psychological disorders than their non-recipient counterparts (OR 2.85, 95% CI 2.07-3.92)
Jayakody et al. (2000) [28]	United States	How does the health of social assistance recipients compare to that of non-recipients?	Descriptive: Cross-Sectional	Psychological disorders	Relative to non-recipients, social assistance recipients reported higher rates of psychological disorders (OR 1.35, *p*<0.05).
Kiely and Butterworth (2013) [29]	Australia	What is the longitudinal association between social assistance recipiency and health?	Descriptive:Longitudinal	General mental health	Movement into social assistance recipiency was associated with worse mental health scores (β=-2.45, *p*<0.001)
Løyland et al. (2011) [30]	Norway	How does the health of social assistance recipients compare to the health of non-recipients?	Descriptive: Cross-Sectional	Psychological symptoms	Relative to non-recipients, social assistance recipients reported a higher frequency of psychological symptoms, including higher rates of sadness, fearfulness, and hopelessness.
Muennig et al. (2013) [36]	United States	How did welfare reform affect the health of social assistance recipients?	Experimental	Mortality	Relative to the non-participant control group, social assistance recipients who participated in the Florida Family Transition Program experienced a 16% higher mortality rate (95% CI 14%-19%).
Narain et al. (2017) [31]	United States	How did welfare reform affect the health of social assistance recipients?	Quasi-Experimental	Self-rated health	Among white low-income single mothers, welfare reform was associated with a 7.0% increase in the prevalence of poor self-rated health (95% CI 1%-12%). Significant estimates were not found among other racial subgroups.
Rodriguez (2001) [32]	GermanyUnited KingdomUnited States	Does the receipt of social assistance mitigate the adverse health consequences of unemployment?	Descriptive: Longitudinal	Self-rated health	Among the unemployed, recipients of social assistance reported higher rates of poor self-rated health relative to non-recipients in Germany (OR 2.23, 95% CI 1.14-4.35), the United Kingdom (OR 1.59, 95% CI 1.08-2.35), and the United States (OR 2.41, 95% CI 1.43-4.06).
Rodriguez et al. (1997) [33]	United States	Does the receipt of social assistance mitigate the adverse health consequences of unemployment?	Descriptive: Cross-Sectional	Depressive symptomsSelf-rated health	Among the unemployed, recipients of social assistance reported a higher frequency of depressive symptoms (β=10.8, 95% CI 5.23-16.2) relative to their non-recipient counterparts.
Rodriguez et al. (2001) [34]	United States	Does the receipt of social assistance mitigate the adverse health consequences of unemployment?	Descriptive: Longitudinal	Depressive symptoms	Among the unemployed, recipients of social assistance reported a higher frequency of depressive symptoms in both the short and long term relative to their non-recipient counterparts.
Vozoris and Tarasuk (2004) [35]	Canada	How does the health of social assistance recipients compare to that of non-recipients?	Descriptive: Cross-Sectional	Chronic conditionsDepressionSelf-rated health	Relative to non-recipients, social assistance recipients reported significantly higher rates of poor self-rated health (OR 3.9, 95% CI 2.8-5.3), depression (OR 2.7, 95% CI 1.9-4.0), diabetes (OR 2.4, 95% CI 1.3-4.4), and obesity (OR 1.6, 95% CI 1.1-2.3).
Wilde et al. (2014) [37]	United States	How did welfare reform affect the health of social assistance recipients?	Experimental	Mortality	Relative to the non-participant control group, social assistance recipients who participated in the Connecticut Jobs First initiative reported a sizeable though statistically insignificant increase in mortality (OR 1.13, 95% CI 0.87-1.46).

### Data sources and population characteristics

Most of the studies involved secondary analyses of nationally representative survey data [22–35]. Two relied on population-based administrative data [36, 37]. A final study drew from a smaller community cohort study [38]. With respect to study populations, ten of the studies looked at the general working-age population [22, 24, 25, 27, 29, 30, 32–35]. Another focused only on women [26]. Two restricted their analyses to socioeconomically disadvantaged individuals within the working-age population [36, 37]. The other four further restricted their analyses to socioeconomically disadvantaged women [23, 28, 31, 38]. The studies showed a high degree of geographic concentration. Nine of the seventeen studies were based in the United States [23, 26, 28, 31, 33, 34, 36–38]. Of the remaining eight studies, five were situated in other English-speaking liberal political economies characterized by weakly redistributive social policies, Australia, Canada, and the United Kingdom [24, 25, 27, 29, 35]. Two other single-country studies examined data from Norway and Sweden [22, 30]. Finally, one cross-national case study compared data from Germany, the United Kingdom, and the United States [32].

### Policy exposures

Seven studies compared the health of social assistance recipients to that of their non-recipient counterparts in Canada, Norway, Sweden, and the United States [22, 24, 25, 28, 30, 35, 38]. Another two examined the health impact of transitions in and out of social assistance recipiency [26, 29]. Four studies measured the health impact of change in the coverage or generosity of social assistance programs in the United States, also known as welfare reform [23, 31, 36, 37]. Welfare reform ended guaranteed federal income support to poor families with children. They also imposed a lifetime limit on the receipt of public assistance and introduced new work-related eligibility requirements [39, 40]. Of the four studies examining the health impact of welfare reform, two looked at the 1996 Personal Responsibility and Work Opportunity Reconciliation Act (PRWORA) [23, 31], another looked at the 1994 Florida Family Transition Program (FFTP) [36], and the last looked at the 1996 Connecticut Jobs First (CJF) initiative [37]. The final four studies assessed whether social assistance mitigates the adverse health consequences of unemployment by comparing jobless recipients and non-recipients [27, 32–34].

### Study designs

Eight studies drew on a descriptive cross-sectional research design [22, 24, 25, 27–30, 33, 35]. Another five studies employed a descriptive longitudinal research design [26, 29, 32, 34, 38]. The final four studies exploited natural policy experiments to estimate the health impact of welfare reform. Two of these constructed quasi-experiments using difference-in-differences and synthetic control designs to compare change in the health status of policy-exposed and policy-unexposed groups before and after the implementation of PRWORA in the United States [23, 31]. Due to data limitations, neither of these quasi-experimental studies could identify those who were directly affected by welfare reform. Rather, in both cases, the treatment group consisted of those the authors believed were most likely to have been affected; namely, socioeconomically disadvantaged single mothers. The final two papers used policy experiments in Florida and Connecticut to examine the impact of welfare reform [36, 37]. Specifically, the authors compared mortality rates between a treatment group that participated in the reformed social assistance program and a control group that retained their traditional benefits.

### Outcomes

Most of the seventeen studies investigated more than one relevant health outcome. More than half of the studies examined the impact of social assistance on one or more dimensions of mental health, including depression, common mental disorders, and adverse psychological symptoms [22, 24–30, 33–35, 38]. Five studies included self-rated health as an outcome [31–33, 35, 38]. Three explored health behaviours such as smoking, drinking, and diet [22, 23, 26]. Two studies focused on mortality [36, 37]. Another two looked at chronic conditions and major risk factors for disease such as hypertension and diabetes [35, 38].

### Findings

All thirteen descriptive studies found that social assistance was associated with adverse health outcomes. Six cross-sectional studies (quality: weak) comparing the health of social assistance recipients to that of the general population found that recipients reported worse health outcomes than their non-recipient counterparts, even after adjusting for key confounders [22, 24, 25, 28, 30, 35]. In Australia, Canada, Sweden, Norway, and the United States, social assistance recipients reported higher levels of adverse psychological outcomes. The Canadian study also observed an association between social assistance recipiency and higher rates of poor self-rated health (odds ratio (OR) 3.9, 95% confidence interval (CI) 2.8–5.3). The Swedish and American studies found worse health behaviours among social assistance recipients, including higher rates of smoking, binge drinking, and harmful dietary habits. In Sweden, for example, the odds of smoking were 4.59 (95% CI 3.56–5.93) times higher among social assistance recipients. Another four descriptive studies spanning three countries (quality: weak or moderate) examined the role of social assistance as a buffer against the adverse health consequences of unemployment [27, 32–34]. All four studies failed to identify a protective effect. Rather, they found that those who were unemployed and receiving social assistance reported worse self-rated health, a greater frequency of depressive symptoms, and a higher rate of psychological disorders. Of the remaining descriptive studies, one longitudinal analysis (quality: weak) of a small cohort study in United States reported an association between the receipt of social assistance during young or middle adulthood and adverse health outcomes twenty or thirty years later, including higher rates of poor self-rated health (OR 2.51, *p* < 0.05, no confidence intervals reported) [38]. The final two descriptive studies (quality: strong) investigated transitions in and out of social assistance in Australia and the United States and found that a movement into social assistance was associated with a higher frequency of depressive symptoms (β = 0.06, *p* < 0.05), worse mental health scores (β = − 2.45, *p* < 0.001), and higher rates of binge drinking (OR 2.06, *p* < 0.05, no confidence intervals reported) [26, 29].

All four experimental or quasi-experimental studies examining the health impact of welfare reform in the United States found that such reforms were associated with adverse health trends. Two studies examined the effects of PRWORA (quality: strong) and found that welfare reform was associated with a 7% increase in the prevalence of poor self-rated health (95% CI 1–12%), an 8.8% increase in the prevalence of smoking (95% CI 6.8–10.8%), and an 8.3% increase in the prevalence of binge drinking (95% CI 14–19%) among the socioeconomically disadvantaged mothers most likely to have been directly affected [23, 31]. Another study (quality: strong) found a 16% (95% CI 14–19%) higher mortality rate among social assistance recipients who participated in the FFTP welfare reform experiment relative to a control group receiving the more traditional and generous set of benefits [36]. A similar investigation of the CJF welfare reform experiment (quality: strong) found higher mortality rates among program participants, though, due in large part to small sample sizes, these estimates did not reach statistical significance [37].

### Quality assessment

The results of the methodological quality assessment are presented in Table 3. Seven studies were deemed to be low quality, four studies were moderate quality, and six studies were strong quality. The most common methodological issue was the absence of an experimental or quasi-experimental study design that is capable, at least to some extent, of controlling for unobserved sources of confounding. Only four of the studies were specifically designed to distinguish true policy effects from potential sources of selection bias that render a comparison of recipients and non-recipients problematic. Furthermore, though many of the studies controlled for the most common confounders (e.g. age, gender, marital status, household size, education), few explicitly accounted for the fact that a significant majority of non-recipients are, by definition, ineligible for social assistance (e.g. due to incomes above means-test thresholds) and therefore serve as inappropriate controls.

**Table 3:** Quality assessment of the studies included in the review

Article	Global Rating	Sample Representativeness	Study Design	Sample Description	Confounding	Attrition
Baigi et al. (2008) [22]	Weak	Strong	Weak	Weak	Weak	N/A
Basu et al (2016) [23]	Strong	Strong	Strong	Strong	Strong	N/A
Butterworth (2003) [24]	Moderate	Strong	Weak	Strong	Strong	N/A
Butterworth et a.l (2011) [25]	Weak	Strong	Weak	Weak	Strong	N/A
Dooley and Prause (2002) [26]	Strong	Strong	Moderate	Strong	Strong	Strong
Ensminger and Juan (2001) [38]	Weak	Weak	Moderate	Weak	Strong	Strong
Ford et al. (2010) [27]	Weak	Strong	Weak	Weak	Weak	N/A
Jayakody et al. (2000) [28]	Weak	Strong	Weak	Weak	Strong	N/A
Kiely and Butterworth (2013) [29]	Strong	Strong	Moderate	Strong	Strong	Strong
Løyland et al. (2011) [30]	Weak	Weak	Weak	Strong	Weak	N/A
Muennig et a.l (2013) [36]	Strong	Strong	Strong	Moderate	Strong	N/A
Narain et al. (2017) [31]	Strong	Strong	Strong	Strong	Strong	N/A
Rodriguez et al. (1997) [33]	Moderate	Strong	Weak	Strong	Strong	N/A
Rodriguez (2001) [32]	Moderate	Strong	Moderate	Weak	Strong	Moderate
Rodriguez et a.l (2001) [34]	Moderate	Strong	Moderate	Weak	Strong	Moderate
Vozoris and Tarasuk (2004) [35]	Weak	Strong	Weak	Strong	Weak	N/A
Wilde et al. (2014) [37]	Strong	Strong	Strong	Strong	Strong	N/A

## Discussion

There are several important insights to be gained from our systematic review. Most notably, the results of our review suggest that social assistance recipients tend to exhibit worse health outcomes relative to their non-recipient counterparts. This appears to be the case even after controlling for key demographic and socioeconomic characteristics. This is somewhat puzzling, given that public health theory would predict that these programs are beneficial to health status [1, 9]. The observation that those receiving benefits are faring worse than seemingly comparable non-recipients may reflect that there are, in fact, systematic differences between these populations that are not readily observable using the data upon which these studies rely. There are least two major sources of confounding that could be biasing the results of the reviewed studies. Firstly, individuals who suffer from pre-existing health problems may be selecting into social assistance programs as a means of accessing ancillary benefits that are otherwise out of reach (e.g. health insurance coverage). Secondly, pre-existing health problems may contribute to adverse socioeconomic experiences such as job loss which in turn predict social assistance status. Indeed, there is evidence suggesting that those who suffer from psychological problems have a greater likelihood of experiencing socioeconomic disadvantage and selecting into social assistance [41–43]. In a similar vein, findings from the extant literature indicate that problematic risk behaviours such as binge drinking may predict later life socioeconomic hardship, thereby influencing social assistance status [44, 45]. In addition, individuals with unreported material resources such as savings and family wealth may be opting out of, or be ineligible for, these benefits. In all three cases, these residual sources of confounding are likely to bias results towards a negative association between income support and health status.

Alternatively, these findings may reflect the fact that social assistance is increasingly conditional on a range of punitive, work-related obligations that compel entry into precarious employment conditions [46–48]. While these measures have been shown to marginally improve employment outcomes among welfare recipients, the terms of their attachment to the labour market tend to be short-lived and produce their own set of adverse socioeconomic consequences, including higher rates of in-work poverty [49]. In fact, recent evidence suggests that these precarious working conditions may pose an equal if not greater risk to health status than the experience of unemployment [50–52]. Based on these findings, we might expect social assistance programs that compel marginal labour market attachment to produce negligible or even negative returns to health. Indeed, evidence from the broader literature demonstrates that alternative income maintenance programs which place fewer behavioural requirements on recipients and provide more generous benefit levels than social assistance programs (e.g. unemployment benefits, earned income tax credits, and unconditional cash transfers) have a positive effect on individual health [53–57]. The finding here that social assistance programs are not similarly associated with positive health outcomes may reflect that, unlike other forms of income maintenance, the scope and generosity of existing social assistance programs are insufficient to offset the negative health consequences of the severe socioeconomic disadvantage that renders one eligible for such programs.

In contrast to the puzzling findings reported in descriptive studies, evidence from experimental and quasi-experimental studies of welfare reform in the United States conform to our theoretical expectations. When benefits were reduced and work conditionalities were intensified, there were observable declines in the health status of the socioeconomically disadvantaged groups who tend to be the principal recipients of welfare; namely, poor and low-educated single mothers. Welfare reform is often assumed to promote work and earnings by encouraging reattachment to the labour market. However, the results of existing evaluations suggest that these returns are lukewarm at best [58, 59]. Furthermore, many households affected by welfare reform experienced heightened levels of material hardship [60]. Often, this was because women who were forced to leave welfare ended up in low-paying, insecure jobs [61]. The results of our systematic review lend support to this view by demonstrating that these reforms have had a negative impact on health status, an outcome that is sensitive to material conditions. Thus, while the main finding that social assistance programs do not appear to be succeeding at maintaining the health of the poor frustrates prevailing public health theory, our review provides some evidence suggesting that a reversal of these earlier welfare reforms and a resulting increase in the scope and generosity of social assistance benefits may have a positive effect on the health of socioeconomically disadvantaged populations.

There are several limitations to our analysis. First, we were not able to identify and include studies evaluating policy experiments involving the expansion of social assistance programs. Policy reforms in high-income countries have overwhelmingly involved the retrenchment of established levels of social protection [62–64]. Consequently, there are few examples of expansionary policymaking available for evaluation. Second, we restricted our search to peer-reviewed journal articles. Evidence collected in books, reports, and working papers were excluded from the review. We also restricted our search to English-language publications. This may explain why most of the studies included in the review were from English-speaking countries characterized by relatively weak welfare state infrastructures, with a majority being from the United States. Finally, due to heterogeneity across studies both in policy exposures and health outcomes, we were not able to conduct a meta-analysis of their results.

## Conclusions

The overall results of our systematic review suggest that evidence on the health impacts of social assistance remains patchy. Rigorous evaluations of these programs are particularly lacking. Few of the studies accounted for systematic differences between social assistance recipients and their non-recipient counterparts. Fewer still adopted the strongest available methods and study designs to evaluate the health effects of policies. We believe there are several principal reasons for the lack of available evidence on the question examined in this review. It may be the case that existing sources of data provide insufficient information for the conduct of rigorous policy evaluations. For example, population-based health surveys tend to provide little if any information on the benefit characteristics of respondents. In addition, while those working in the field of public health may be increasingly familiar with appropriate statistical techniques to evaluate societal-level policy interventions [65–67], social assistance programs may not be particularly amenable to the application of such methods. For example, many of the best available methods (e.g. regression discontinuity, difference-in-differences, and interrupted time series designs) require researchers to identify moments of large-scale policy change. In contrast to other areas of public policymaking, such as tobacco or food labelling, social assistance programs are rarely affected by such abrupt punctuations. A notable exception in this regard is welfare reform in the United States, for which there is evidence that we have reviewed here [23, 31, 36, 37]. Finally, institutional barriers associated with the conduct of politically sensitive research may be standing in the way of the generation and dissemination of evidence on social assistance programs. Tackling the structural determinants of health requires large-scale government interventions (e.g. greater income redistribution and labour market regulation) [3]. Such efforts can attract opposition from political actors who oppose such a role for governments [68–74]. Many epidemiologists and other scientists contributing to the health inequalities literature may, in turn, feel that conducting and disseminating research of this nature is too political or, by virtue of the political opposition they believe it might face, too challenging to undertake [75, 76].

Notwithstanding these important challenges, there is a growing need for evidence on the health effects of social assistance and similar social policies [77]. While governments often identify health equity as an important priority, their choice of interventions have largely relied on behavioural health promotion strategies that fail to account for the role of social policies as necessary levers to reduce health inequalities [78, 79]. Because efforts to eliminate or even reduce health inequalities are unlikely to be successful if they fail to intervene upon their fundamental causes, it is imperative that public health researchers examine these policies and identify the structural interventions that hold the greatest (and the least) promise for reducing health inequalities [80]. The paucity of such evidence is particularly problematic in light of growing evidence that, despite more than a decade of efforts to promote health equity, inequalities in major indicators of population health appear to be widening [81, 82]. These troubling findings may reflect underlying changes in the social and economic architectures of high-income countries, such as the retrenchment of social protection policies – including social assistance programs [49, 62, 83] – and concomitant increases in adverse socioeconomic experiences, such as poverty and unemployment [84, 85]. Taken together, these broader trends highlight a continuing need for solid evidence to marshal in support of interventions that target the fundamental determinants of health.

## Additional file

Additional file 1: Modified Quality Assessment Tool for Quantitative Studies. This file provides a detailed description of the tool used to assess the methodological quality of studies included in the systematic review. (DOCX 75 kb)

## Abbreviations

CI: Confidence interval
CJF: Connecticut Jobs First
FFTP: Florida Family Transition Program
OR: Odds ratio
PRISMA: Preferred Reporting Items for Systematic Reviews and Meta-Analyses
PRWORA: Personal Responsibility and Work Opportunity Reconciliation Act
