# Supplementary material for: The impact of social assistance programs on population health: a systematic review of research in high-income countries
Source: BMC Public Health. 2019 Jan 3;19:2. doi: 10.1186/s12889-018-6337-1 (PMC6318923; doi:10.1186/s12889-018-6337-1)
Supplement: Supplementary file 1 — Modified Quality Assessment Tool for Quantitative Studies. This file provides a detailed description of the tool used to assess the methodological quality of studies included in the systematic review. (DOCX 75 kb) [file 12889_2018_6337_MOESM1_ESM.docx]

**Appendix**

Modified Quality Assessment Tool for Quantitative Studies

from the National Collaborating Centre for Methods and Tools [1]

**Component A: Study Representativeness**

Are the individuals selected to participate in the study likely to be representative of the target population?

1. Very likely (strong)
2. Somewhat likely (moderate)
3. Not likely (weak)
4. Can’t tell (weak)

**Component B: Study Design**

Indicate the study design.

1. Randomized controlled trial or experiment (strong)
2. Cohort analytic or quasi-experiment (strong)
3. Descriptive longitudinal (moderate)
4. Descriptive cross-sectional (weak)

**Component C: Sample Description**

Are the demographic and socioeconomic characteristics of the exposed and unexposed individuals described?

1. Yes, in full (strong)
2. Yes, partially (moderate)
3. No (weak)

**Component D: Confounding**

Were there important differences between groups prior to the intervention?

1. Yes
2. No (strong)

If yes, were key sources of confounding such as age, sex, marital status, household structure, education, and baseline health status controlled for?

1. Yes (strong)
2. No (weak)

**Component E: Attrition**

If the study was longitudinal, were attrition rates reported?

1. Yes
2. No (weak)

If yes, indicate the follow-up rate.

1. 80-100% (strong)
2. 60-79% (moderate)
3. Less than 60% (weak)

[1] National Collaborating Centre for Methods and Tools. *Quality assessment tool for quantitative studies.* Hamilton, ON: McMaster University, 2008.
